# Declining burden of malaria over two decades in a rural community of Muheza district, north-eastern Tanzania

**DOI:** 10.1186/1475-2875-12-338

**Published:** 2013-09-22

**Authors:** Deus S Ishengoma, Bruno P Mmbando, Method D Segeja, Michael Alifrangis, Martha M Lemnge, Ib C Bygbjerg

**Affiliations:** 1 National Institute for Medical Research, Tanga Medical Research Centre, P.O Box 5004, Tanga, Tanzania; 2 Centre for Medical Parasitology at the Department of International Health, Immunology and Microbiology, University of Copenhagen, Copenhagen, Denmark; 3 Department of Infectious Diseases, National University Hospital (Rigshospitalet), Copenhagen, Denmark

## Abstract

**Background:**

The recently reported declining burden of malaria in some African countries has been attributed to scaling-up of different interventions although in some areas, these changes started before implementation of major interventions. This study assessed the long-term trends of malaria burden for 20 years (1992–2012) in Magoda and for 15 years in Mpapayu village of Muheza district, north-eastern Tanzania, in relation to different interventions as well as changing national malaria control policies.

**Methods:**

Repeated cross-sectional surveys recruited individuals aged 0 – 19 years from the two villages whereby blood smears were collected for detection of malaria parasites by microscopy. Prevalence of *Plasmodium falciparum* infections and other indices of malaria burden (prevalence of anaemia, splenomegaly and gametocytes) were compared across the years and between the study villages. Major interventions deployed including a mobile clinic, bed nets and other research activities, and changes in national malaria control policies were also marked.

**Results:**

In Magoda, the prevalence of *P. falciparum* infections initially decreased between 1992 and 1996 (from 83.5 to 62.0%), stabilized between 1996 and 1997, and further declined to 34.4% in 2004. A temporary increase between 2004 and 2008 was followed by a progressive decline to 7.2% in 2012, which is more than 10-fold decrease since 1992. In Mpapayu (from 1998), the highest prevalence was 81.5% in 1999 and it decreased to 25% in 2004. After a slight increase in 2008, a steady decline followed, reaching <5% from 2011 onwards. Bed net usage was high in both villages from 1999 to 2004 (≥88%) but it decreased between 2008 and 2012 (range, 28% - 68%). After adjusting for the effects of bed nets, age, fever and year of study, the risk of *P. falciparum* infections decreased significantly by ≥97% in both villages between 1999 and 2012 (p < 0.001). The prevalence of splenomegaly (>40% to <1%) and gametocytes (23% to <1%) also decreased in both villages.

**Discussion and conclusions:**

A remarkable decline in the burden of malaria occurred between 1992 and 2012 and the initial decline (1992 – 2004) was most likely due to deployment of interventions, such as bed nets, and better services through research activities. Apart from changes of drug policies, the steady decline observed from 2008 occurred when bed net coverage was low suggesting that other factors contributed to the most recent pattern. These results suggest that continued monitoring is required to determine causes of the changing malaria epidemiology and also to monitor the progress towards maintaining low malaria transmission and reaching related millennium development goals.

## Background

Malaria has been a major cause of morbidity and mortality in tropical areas particularly in sub-Saharan Africa (SSA), and was estimated to cause 300 – 350 million cases and 0.5 - 1 million deaths annually between 2000 and 2007 [1]. However, recent reports have shown that the burden of malaria has declined in several countries with remarkable differences between different regions of Africa [1,2]. In southern Africa, a significant decline of malaria burden has been reported in the South African Republic [3-6], Zambia [7,8], Swaziland and Southern part of Mozambique [9,10]. A similar decline has also been reported in the Horn of Africa (Ethiopia and Eritrea) [11-15] and in isolated islands of Zanzibar [16,17], Sao Tome and Principe [18] and Bioko Island in Equatorial Guinea [19,20]. Reports from other parts of Africa show inconsistent results, although a declining burden of malaria has been reported in some countries, including Kenya [21-24], Tanzania [25-28], Rwanda [15,29], the Gambia [30,31] and Gabon [32]. These changes have been attributed to scaling-up of control interventions such as deployment of artemisinin-based combination therapy (ACT), insecticide-treated bed nets (ITNs), indoor residual spraying (IRS) and larval control [2,33]; yet, there are indications that the declining malaria burden in some of the countries actually started before implementation of such major intervention programmes [2]. A recent study conducted in Kenya revealed that drivers of changing malaria epidemiology in some areas might not be entirely attributed to claimed interventions [34].

In Tanzania, malaria has until the mid-2000s been responsible for over 18 million cases, 100,000 deaths/year and accounting for over 43% of outpatient cases, 30% of all hospital admissions and 40% of deaths occurring at health facilities [35]. Despite few reports which have indicated that malaria has declined in most parts of the country [36], most of the data obtained from health facilities through the health management information system (HMIS) have revealed that the burden of malaria in Tanzania has remained largely unchanged with high and similar proportions of hospital attendances, admissions and deaths between 2003 and 2010, as documented in the World Health Organization (WHO) report of 2011 [1]. However, the most recent Tanzania HIV and malaria indicator surveys (THMIS) conducted in 2011/2012 have shown that malaria parasite prevalence has declined by >50% since the latest surveys of 2007 (from 18% to 9%), with the highest parasite prevalence of 33% in the North-western region of Geita near Lake Victoria and the lowest of <1% in several regions of North-western, Central, Southern highlands and Zanzibar Island (MoH, unpublished data). Furthermore, studies conducted in Muheza district of north-eastern Tanzania have showed a concurrent decline of malaria incidence among hospitalized children, and interestingly, also of community acquired bacteraemia, suggesting that the decline in malaria burden is also affecting trends of bacterial infections [26].

The data used in the WHO report [1] were derived from hospital records through HMIS which in most cases are over-estimated by including majority of patients diagnosed based on symptoms only. Even in areas with the capacity for malaria diagnosis by microscopy, previous studies showed that malaria was commonly over-diagnosed especially in areas of low transmission, mainly attributed to limited skills of microscopists for preparation and examination of blood smears [37]. Thus, more detailed studies are required to determine the current malaria burden in Tanzania particularly after introduction of malaria rapid diagnostic tests (RDTs) in 2012.

Apart from the mentioned national surveys, the observed decline in the burden of malaria in Tanzania is based on few detailed studies [25-28] and thus, there is limited information from other malaria endemic areas in the country. Furthermore, the reasons for the decline of malaria burden in these particular areas are not clear, although some of the interventions deployed in recent years are believed to be the main cause of the current changes in malaria epidemiology [1,25,27]. This indicates that more detailed studies are required to document the trend of malaria in areas with variable endemicity and to provide reliable information on the possible association between interventions in place and resulting effects on malaria burden, for supporting formulation of new policies for malaria control. Finally, the current decline in the burden of malaria, when confirmed, will demand for a significant changes in malaria control to identify and treat the remaining cases and eventually attempt to block malaria transmission as well as improve management of non-malaria fevers.

A long-term presence in the two rural villages of Muheza district enabled the present study to assess the long-term trends of malaria burden over a period of 20 years (1992 – 2012). In these villages, which were until recently holo/hyper-endemic to malaria, different inventions (including ITNs, passive case detection and treatment of malaria, and different anti-malarial drug trials) have been deployed, reflecting changing local demands and national policies. Their potential contribution to the observed trends of decreasing malaria is discussed, while the remarkable changes of parasite prevalence are given in more details, including differences between the two nearby villages.

## Methods

### Study site and interventions between 1992 and 2012

This study utilized assembled data collected during repeated cross-section surveys (CSS) conducted between September 1992 and June 2012 in the village of Magoda and between 1997 and June 2012 in the neighbouring village of Mpapayu both in Muheza district in Tanga region, North-eastern Tanzania. The villages were involved in malaria and filariasis studies as described elsewhere [38-40]. Currently, the two villages have a population of 2,934 individuals in 678 households and under-fives account for 13.2% of the entire population. The data generated by previous studies, therefore, provide additional value in assessing the historical evidence to demonstrate the impact of different interventions and policies over the period.

After an initial screening of parasite prevalence of the entire population of Magoda village in 1992, and based on the promising results from the Gambia [41], a Maloprim^®^ (3.125 mg pyrimethamine and 25 mg dapsone, GlaxoSmithKline, UK) trial was the first study to be implemented in Magoda in 1993–94 to test the prophylactic effects of weekly administration of the drug on parasite infections, anaemia and splenomegaly [40]. Children aged <10 years old were given either one or two tablet(s) of Maloprim^®^ to under-fives and those aged 5–9 years, respectively and followed-up weekly to determine their general condition and malaria infection status [40]. Because of increasing resistance to chloroquine (CQ) in Muheza district which was then the government policy as first line treatment of uncomplicated malaria [42], patients who developed parasitaemia during follow-up under this trial were treated with either sulphadoxine/pyrimethamine (SP) or amodiaquine (AQ) [40].

Subsequently, a mobile clinic, visiting Magoda village twice weekly was introduced in 1994 to estimate if a rebound of malaria may arise after termination of the Maloprim^®^ trial, and the mobile clinic was continued till November 2004 to provide medical services to the community. The mobile services and malaria monitoring were extended to the neighbouring village of Mpapaya from 1997. During the visits by the mobile clinic, suspected cases of malaria had blood smears taken, and treatment was given on clinical grounds using either SP or AQ, because blood smears were only examined after returning to the research laboratory, for practical reasons. Patients not symptomatically treated but with malaria parasites in blood smears, were given antimalarials on the following day after microscopy results were obtained. Furthermore, repeated *in vivo* drug efficacy trials were carried out and involved patients with uncomplicated malaria (confirmed by microscopy) in 1994–1996 [43-45], 1997 [46] and 1998–1999 [38] testing SP, and later AQ in 2003 [47]. In December 1998, each sleeping bed in all households in Magoda (on request from the villagers), received a permethrin-treated net (ITN) while in Mpapayu, deltamethrin-treated nets were distributed in March 2001; all ITNs were re-impregnated twice a year [38] until 2003. Throughout the observation period (up to 2004), children and adults with fever and other ailments requiring hospitalization were offered admission to the local designated district hospital of Muheza located 10 km from the village. By 2005, a local dispensary was built to replace the mobile clinic while artemisinin-combination therapy (ACT, with artemether/lumefantrine, AL) was introduced in January 2007, following changes of malaria treatment policy [48]; and rapid diagnostic test for malaria (RDTs) were introduced in 2012 (NMCP, Personal Communication).

### Recruitment of participants and data collection

For this paper, complete data sets with personal details which were recorded on a morbidity questionnaire including age, sex and residence, parasite infection status for asexual and sexual stages, haemoglobin (Hb) levels, splenomegaly and ITN use and insecticide impregnation status were available for CSS conducted between 1999 and 2012 (Table 1). Additionally, summarized data were retrieved from reports covering the surveys conducted in 1992 [49] and for other years (1995, 1996, 1997 and 1998), quarterly/annual reports of Amani Centre of the National Institute for Medical Research (NIMR) were used to get the prevalence and density of *P. falciparum* infections. The CSS involved individuals aged between six months and 20 years. The surveys were conducted during (April/June) or after (July/September) the long rain season between September 1992 and June 2012 as described elsewhere [38,40,49,50].

**Table 1:** **Prevalence of *P. falciparum *gametocytes, splenomegaly and geometric mean gametocyte density among individuals who participated in malaria cross sectional surveys in Magoda and Mpapayu villages in Muheza district from 1999 – 2012**

**Year**	**Magoda**				**Mpapayu**			
	**n (% under-fives)**	**With splenomegaly (%)**	**Pf gametocyte prevalence (%)**	**G-mean Pf gametocyte density**	**n (% under-fives)**	**With splenomegaly (%)**	**Pf. gametocyte prevalence (%)**	**G-mean Pf. gametocyte density**
1999	383 (40.4)	38.6	12.1	31	232 (39.2)	40.5	22	40
2000	352 (27.8)	21.6	6	55	226 (34.5)	43.4	8	85
2001	352 (42.4)	45.6	7.8	95	202 (39.1)	52	5	62
2004	392 (36.0)	23.7	5.6	21	237 (38.7)	23.6	1.7	16
2008	422 (41.2)	0.7	5	32	268 (36.2)	1.6	2.2	49
2009	512 (33.4)	2.5	3.1	23	267 (38.2)	1.9	3	64
2010	480 (33.7)	0.2	0.4	121	273 (34.4)	1.1	0.7	22
2011	482 (26.6)	0.2	0.2	560	259 (28.2)	0.8	0	0
2012	459 (26.0)	0.7	0.4	52	252 (26.6)	0	0.4	32

Pf = *Plasmodium falciparum*, n = number of individuals enrolled in the surveys, % = percent and G-mean = geometric mean.

Each recruited person was examined by a medical doctor and had axillary temperature taken using a digital thermometer, and splenomegaly was assessed as previously described [51]. Blood samples were collected from each of the study participants by venous bleeding or finger prick for parasitological examination and other laboratory analyses. Thick and thin blood smears were prepared and dried in the field, and later brought to the laboratory for further processing. For the surveys conducted before 2004, blood smears from participants with fever (axillary temperature ≥37.5°C) were examined after the end of each day and those with malaria parasites were treated with SP or AQ on the following day. For the CSS conducted between 2008 and 2012, all participants were tested with RDTs and those with positive test results and other symptoms of uncomplicated malaria were treated with AL as described by Ishengoma *et al.*[50]. Asymptomatic individuals with positive RDT results were not treated with anti-malarials, but were advised to report to the nearest dispensary in Magoda in case they fell sick.

### Laboratory analysis

Blood smears collected in the CSS were stained using 10% Giemsa solution for 30 minutes and examined at a magnification of 1,000× to detect parasite species and to determine parasitaemia. Quality control of results of microscopic examination of blood smears were performed as described elsewhere [50,52]. Briefly, each blood smear was examined by two technicians blinded of the patient status and RDT results. Parasite density was calculated as the average counts of the two technicians if their results did not differ by more than 50% for blood smears with ≥400 asexual parasites/μl of blood. For blood smears with < 400 asexual parasites/μl, any counts of each of the two technicians was accepted and used to calculate the average parasite density. Blood smears with discordant results were re-examined by a third technician and the results of any two technicians was accepted as explained above. Further discordant smears were resolved by a team of three technicians who re-examined such smears at the same time. For the studies done between 1999 and 2004, haemoglobin (Hb) levels were measured by PCV and later converted into Hb levels (g/dl) as described elsewhere [53]. In the CSS conducted from 2008 to 2012, Hb was measured using a HemoCue (HemoCue, Ångelholm, Sweden) and anaemia was described as Hb < 11 g/dl.

### Ethical considerations

The studies which provided data for this paper were approved by the Medical Research Coordination Committee of NIMR. Verbal and written informed consent was sought from patients or parents/guardians in case of children. Village meetings were held to explain and discuss the study plans with community members and feedback including results of previous surveys was given to the communities through the above meetings together with a written report.

### Data analysis

Microsoft Access database was utilized for data management with double entry, validation and cleaning of data collected from 2008 to 2012; followed by analysis using STATA version 11 (STATA Corp Inc., TX, USA). Data collected in 1999–2004 were managed using Epi-Info (CDC, Atlanta, USA) and Microsoft Excel, and later transferred to STATA version 11 (STATA Corp Inc., TX, USA) for analysis. The analysis involved comparison of different variables such as parasite positivity, parasite density, gametocyte carriage, gametocyte density, splenomegaly, mean Hb, anaemia and ITN use in order to test for the differences across years and between the two villages. Categorical data were compared using chi-square test while continuous variables were tested using Students t-test. Logistic regression analysis was used to assess the effects of different interventions (using data collected from 1999 to 2012), such as deployment and distribution of ITNs, anti-malarial drug trials and changes of malaria treatment policy (chloroquine in 1999 – 2001, SP from 2001 to 2004 and AL in 2008 – 2012) on the risk of being infected with malaria parasites after adjusting for age and fever status (if study participants had fever or not). The different interventions which had specific point of deployment such as use of ITNs were coded as binary variables and interventions which covered a particular period were similarly labeled with reference to the duration of such interventions; and these variables were used in logistic regression analysis. Non-normal continuous variables such as parasite density were log transformed to normality. Linear regression models were used in modeling the relationship between Hb concentration and parasite density as response variables against explanatory variables. P-value <0.05 was considered significant.

## Results

### Overall prevalence of *P. falciparum* infections in Magoda (1992–2012) and Mpapayu village (1998–2012)

Although cross-sectional surveys (CSS) were conducted in Magoda from 1992 to 2012, and in Mpapayu village from 1998 to 2012 and involved individuals aged <20 years, complete data (with demographic information, clinical, parasitological and ITNs data) were only available for CSS done between 1999 and 2012 (Table 1). For the period between 1992 and 1998, summarized reports were used to extract the prevalence and density of *P. falciparum* infections obtained in those surveys. For the nine CSS conducted in both villages between 1999 and 2012, a total of 6,050 individuals aged 0–19 years (mean age = 7.8 ± SD 4.7 years) were enrolled (Table 1). Children less than five years of age were 2,111 (34.9%), and 2,984 (49.3%) were males.

**Figure 1:**
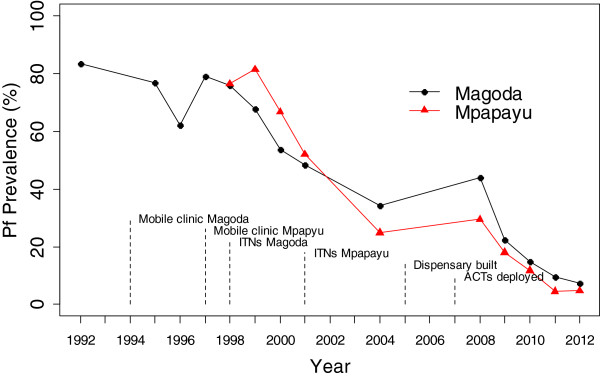
**Overall prevalence of *Plasmodium falciparum *infections and interventions deployed in Magoda and Mpapayu villages in Muheza district from 1992 to 2012.**

In Magoda village studied over twenty years from1992 to 2012, the prevalence of *P. falciparum* infections in all individuals below 20 years of age was 83.5% in 1992 and 88.0% in 1995; with a decline to 62.2% in 1996. This was followed by a slight increase between 1996 and 1997, and a steep decline from 1999 to 2004 (from 67.9% to 34.4%, p < 0.001) (Figure 1). Between 2004 and 2008, a significant increase in *P. falciparum* prevalence occurred (34.4 to 44.0%, p = 0.006), followed by a steep and steady decline to 7.2% in 2012. In the village of Mpapayu, the highest prevalence of *P. falciparum* infections was 81.5% in 1999 and it decreased significantly to 24.9% in 2004 (p < 0.001), followed by a slight increase in 2008, and a steady decline to less than 5% from 2011 onwards (Figure 1).

### Comparison of the *P. falciparum* prevalence between Magoda and Mpapayu villages (1999–2012)

The prevalence of *P. falciparum* was significantly higher in Mpapayu compared to Magoda in 1999 and 2000 (p ≤ 0.002 for all comparisons) while for the CSS conducted in 2004 and 2008 – 2012, the prevalence was significantly higher in Magoda than Mpapayu village (p ≤ 0.018) (Figure 1). After adjusting for the effects of bed nets, age of study participants, fever status, changes in malaria treatment guidelines and year of study (to account for the changes in transmission and different interventions deployed in the study villages), the risk of carrying malaria parasites decreased significantly by 75% between 1999 and 2004 and by 97% between 1999 and 2012 in Magoda village; and by 89% and 99% in Mpapayu over the same periods (p < 0.001, for all comparisons).

### Age-specific prevalence of *P. falciparum* infections in Magoda (1992 – 2012) and Mpapayu village (1999–2012)

**Figure 2:**
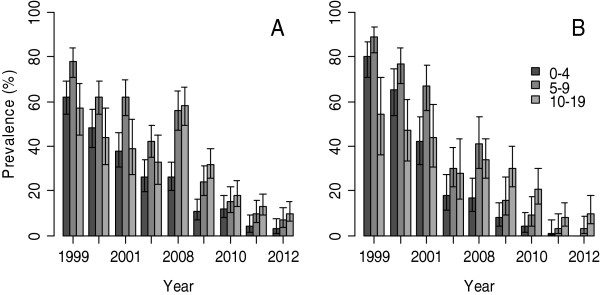
**Age-specific prevalence of *Plasmodium falciparum *infections in the villages of Magoda (A) and Mpapayu (B) between 1999 and 2012.** Confidence intervals were calculated by Wilson score method.

In under-fives, the prevalence of *P. falciparum* infections declined steadily over the years from 83.6% to 26% in Magoda from 1992 – 2004, and 80% to 18.5% in Mpapayu village between 1999 and 2004. Although the overall prevalence did not change between 2004 and 2008 in both villages, it decreased in under-fives from 26% to 3% and 17% to 0% between 2008 and 2012 in Magoda and Mpapayu villages, respectively (Figure 2A and B). Between the villages, the prevalence in under-fives was significantly higher in Mpapayu compared to Magoda in 1999 (p = 0.003) and 2000 (p = 0.015), but was higher in Magoda compared to Mpapayu in 2010.

For individuals aged ≥5 years from Magoda, the prevalence dropped from 91% to 39% between 1992 and 2004, and then increased to 56.7% in 2008, followed by a steady decline to 8.6% in 2012. A similar trend was observed in Mpapayu village. However, the prevalence in the same age group was significantly higher in Mpapayu compared to Magoda in 1999 (p = 0.024) and 2000 (p = 0.043), while it was significantly higher in Magoda in 2004 (p = 0.040), 2008 (p < 0.001) and 2011 (p = 0.034). After adjusting for the effect of the year of survey, the decline in the prevalence of *P. falciparum* infections was higher among under-fives compared to individuals aged ≥5 years in both villages (OR =1.97, 95% CI = 1.72 – 2.24; p <0.001). When the analysis was performed by categorizing study participants into three age groups (0–4, 5–9 and 10–19 years), the prevalence of *P. falciparum* infections in both villages (Magoda and Mpapayu) decreased in all groups across the years with the lowest prevalence observed in 2012. However, the peak parasite prevalence which was observed in children aged 5–9 years up to 2004 shifted to participants aged 10–19 years from 2008 in Magoda and from 2009 onwards in Mpapayu (Figure 2A and B).

### Overall and age-specific geometric mean density of *P. falciparum* infections in Magoda and Mpapayu villages (1999–2012)

In Magoda, the geometric mean parasite density was similar in the surveys conducted between 1999 and 2004 while a significant increase occurred between 2004 and 2008 (p = 0.004); followed by similar parasite density in 2008–2011, and a significant decline in 2012 (p < 0.001). For Mpapayu, the parasite density was similar in 1999 – 2000, followed by a significant decline between 2001 and 2004 (p = 0.008) and a significant increase which was observed between 2004 and 2008 (p = 0.041). The parasite density was similar for the surveys conducted between 2008 and 2011 in Mpapayu, followed by a significant decline in 2012 (p = 0.004) as also observed in Magoda.

**Figure 3:**
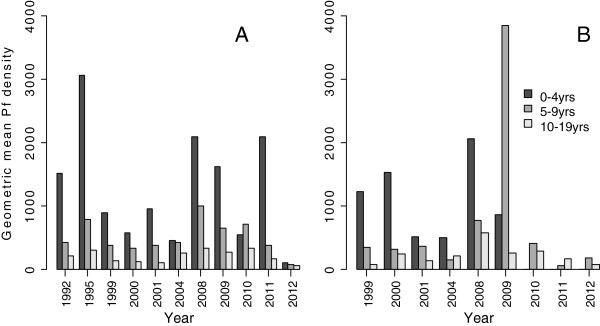
**Age-specific geometric mean of *Plasmodium falciparum *density (asexual parasites/μl) in the villages of Magoda (A) and Mpapayu (B) between 1999 and 2012.** Data of under-fives from Mpapayu village **(B)** in 2010 and 2011 were excluded from the analysis because individuals seen were few and had high parasite density (4 with geometric mean parasite density of 21,744 and one participant with 31,200 asexual parasites/μl, respectively). In Mpapayu, no study participant aged <5 years old had malaria parasites in 2012.

The age-specific geometric mean parasite density was generally higher in under-fives than other age groups and ranged from 107 to 2,093 asexual parasites/μl in Magoda, and 0 to 2,062 asexual parasites/μl in Mpapayu (Figure 3A and B). Data of under-fives from Mpapayu village in 2010 and 2011 were excluded from the analysis and Figure 3B, because individuals seen were few and had abnormally high parasite density (4 with geometric mean parasite density of 21,744 and one participant with 31,200 asexual parasites/μl, respectively). Generally, individuals in all age groups had higher parasite densities in 2008–2011 in Magoda and 2008–2009 in Mpapayu compared to previous CSS and those done in 2012 (Figure 3A and B).

### Mean haemoglobin levels and anaemia in Magoda and Mpapayu villages (1998–2012)

Complete data of haemoglobin levels (Hb, g/dl) were available in under-fives only for the CSS conducted from 1998 to 2000, and between 2008 and 2012. For CSS conducted between 1998 and 2000 in both villages, linear regression model adjusted for the effect of age, gender and parasite density showed that the mean Hb was increasing across the year by 0.18 (95% CI: 0.04 - 0.32), p = 0.011, where the baseline mean Hb concentration was 10.20 g/dl (95% CI: 9.97 -10.41). Between 2008 and 2012, there was an insignificant decrease in mean Hb by 0.03 (95% CI = -0.09 - 0.02), p = 0.237).

### Prevalence of splenomegaly in Magoda and Mpapayu villages (1999 – 2012)

The prevalence of splenomegaly in all age groups was higher in the CSS conducted before 2004 with significantly lower prevalence in the CSS conducted from 2008 onwards (2004 *Vs* 2008; χ^2^ = 156.7, p < 0.001). In Magoda village, the prevalence of splenomegaly was similar for under-fives and those aged ≥5 years (p > 0.168), except in 2004 when it was significantly higher in under-fives (p = 0.001) (see Table 1). Mpapayu village had higher prevalence of splenomegaly than Magoda in most years with significantly higher prevalence in under-fives (p < 0.043) in 1999, 2000 and 2012, while it was significantly higher in those aged ≥5 years in 2004 only (χ^2^ = 11.4, p = 0034). The prevalence of splenomegaly among under-fives was significantly higher in Mpapayu compared to Magoda in 1999 (p = 0.006) and 2000 (p < 0.001) only, while in those aged ≥5 years, the prevalence was higher in Mpapayu in 2000 only (p < 0.001). In all age groups, the highest prevalence of splenomegaly (52.0%) was observed in Mpapayu village in 2001.

### Gametocyte prevalence and density in Magoda and Mpapayu villages (1999 – 2012)

Gametocyte prevalence in all age groups were lower in the surveys conducted from 2008–2010 compared to those conducted before 2004 and it decreased significantly over the study period (χ^2^ for trend = 228.3, p < 0.001). When the two villages were compared, the prevalence was significantly higher in Mpapayu than Magoda in 1999 (p < 0.001), and in Magoda than Mpapayu in 2004 (p = 0.017) (Table 1). From 2008 to 2012, few individuals (≤5%) carried gametocytes whereby only one and two individuals from Magoda village had gametocytes in 2011and 2012, respectively. In Mpapayu, no gametocytes were detected in 2011 while only one individual had gametocytes in 2012.

### Bed net usage in Magoda and Mpapayu villages between 1999 and 2012

**Figure 4:**
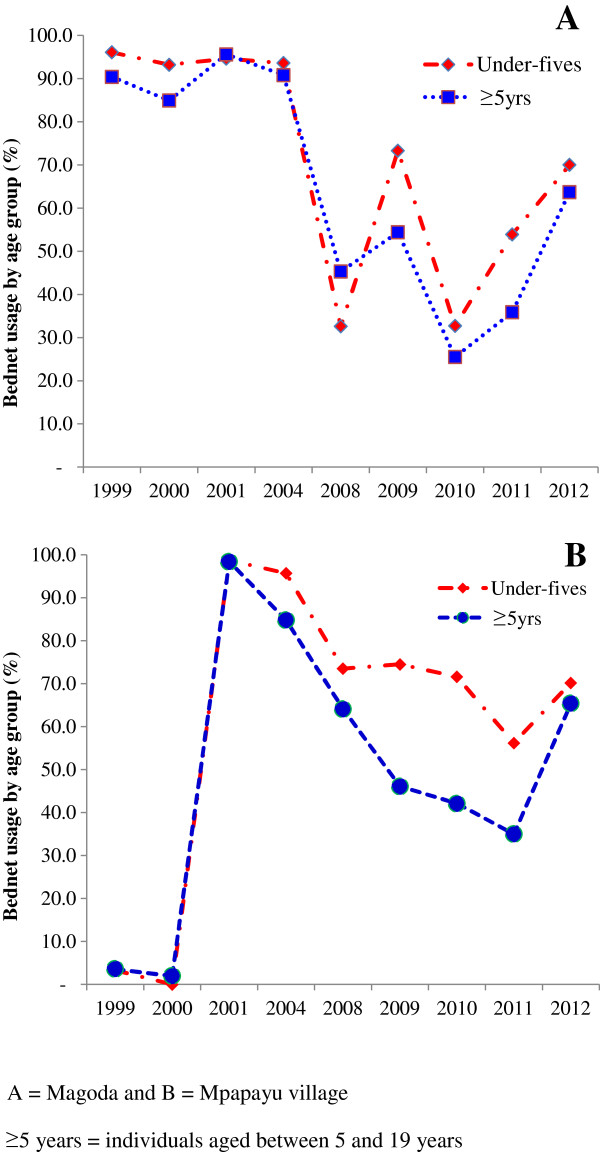
Proportion of under-fives and study participants aged ≥5 years who reported to have slept under bed nets in the past 24 hours before the survey in the villages of Magoda (A) and Mpapayu (B) in Muheza district between 1999 and 2012.

The proportion of individuals who reported to have slept under bed nets a night before the survey (whether impregnated with insecticides or not) was higher in the CSS of 1999 – 2004 than those of 2008–2012, with usage >88% in Magoda between 1999 and 2004 and >90% in Mpapayu between 2001 and 2004 (Figure 4A and B). In Magoda village, individuals (all age groups) using bed nets in 2008 had decreased to ≤45.0% followed by an increase to ≥54.0% in 2009 (Figure 4A). In Mpapayu, very few individuals (<4%) were using bed nets before free ITNs were distributed to all people in 2001. However, the number of individuals using bed nets in Mpapayu increased to ≥85% by 2004 and later decreased to <74% in 2008, and continued to decline reaching the lowest number of users in 2011 (35.0% among individuals aged ≥5 years). In 2012, the number of bed net users increased to over 64.0% in both villages following a national campaign of universal bed net coverage (Figure 4A and B). For the two villages and in all age groups, there were significantly higher bed net usage in Magoda village than Mpapayu in 1999 and 2000 (p < 0.001) while the number of bed net users was significantly higher in Mpapayu in 2008 and 2010 (p < 0.001). The risk of being infected by malaria parasites was significantly higher among individuals not using bed nets in both villages, after adjusting for age, fever status and year of study (Magoda, OR = 1.22, p = 0.044 and in Mpapayu, OR = 1.59, p = 0.003). Over the years, individuals using bed nets in both villages had lower risk of carrying malaria parasites, e.g. by <45% in 2000 and decreased by >97% between 1999 and 2012 (p < 0.001 for all comparisons).

## Discussion

Although the information on the current burden of malaria in most of the malaria endemic countries is fragmented or lacking, there is clear evidence that the burden of malaria has declined in some endemic countries [2]. For Tanzania, a significant decline of malaria burden has been confirmed on the islands of Zanzibar after deployment of intensive malaria interventions including ACT, ITNs and IRS [16,17], whereas data on the changing epidemiology of malaria on the mainland are less well documented and relatively few reports exist except from some parts including north-eastern Tanzania. Furthermore, previous [28] and the most recent Tanzania HIV and malaria indicator surveys (THMIS) of 2011/2012 showed a significant decline of malaria burden, whereby the average parasite prevalence in under-fives decreased from 18% to 9% between 2007 and 2011 (MoH, unpublished data). These nation-wide surveys are aggregated at the regional level and cannot give detailed patterns of malaria at small geographic coverage such as the district.

Studies of malaria (and other parasitic diseases, such as lymphatic filariasis) including repeated cross-sectional studies were conducted in the villages of Magoda (since 1992) and Mpapayu (since 1997) in Muheza district of north-eastern Tanzania and have enabled long-term observations of trends in malaria for over 15–20 years. The studies also showed the possible impact of the deployed interventions at the community level and changing national malaria control policies. These villages and other parts of Muheza have been reported to have high burden of malaria, as well as high levels of resistance to different anti-malarial drugs [43-47]. Treatment-wise, in Magoda village, SP and AQ were deployed early in 1994 during the Maloprim^®^ trial [40], and CQ was withdrawn (due to high level of resistance) even before the official change of malaria treatment policy replacing CQ with SP were effected in 2001 [54].

Overall, the study reported here observed a significant decline of malaria burden over the study period. Although the major decline in the prevalence of *P. falciparum* infections occurred between 1997 and 2004 (Magoda) and 1998–2004 (Mpapayu) when more interventions were being implemented, further changes were also observed between 2008 and 2012 in both villages. The peak prevalence of malaria shifted from children aged 5–9 to those aged 10–19 years old in 2008–2012, a trend also observed in the highlands of the neighbouring district of Korogwe which generally have low malaria transmission [51]. The prevalence of splenomegaly and gametocytes also declined during the same period.

The decline in prevalence observed before 1999 could possibly be due to better services provided by the research team while the changes observed between 1999 and 2004 could also be attributed to drug trials, which were implemented from 1994 [43-47], and later deployment of free ITNs [38]. During these trials, the research teams were always available in the communities and provided health services to other patients including treatment of malaria, regardless of their involvement in the studies. The deployment of ITNs at different time points in the two villages, while other services were otherwise equal, enabled an estimation of the impact of ITNs on malaria and prevalence of drug resistance markers [38]. High bed net coverage has also been associated with reduced malaria morbidity and mortality as reported in previous studies [55,56].

Presence of research team in the villages, deployment of a mobile clinic and other services provided by village health workers would also possibly reduce malaria burden through prompt diagnosis and treatment and referral to Muheza district hospital for free services, when needed. Furthermore, the records collected between 1992 and 2004 showed that the number of deaths declined significantly and cerebral malaria virtually disappeared from Magoda and Mpapayu village (Ib C. Bygbjerg, personal communication). Similar studies conducted in the neighbouring district of Korogwe also showed that improved health services at the community level through deployment and use of village health workers under the supervision of trained medical personnel was associated with a significant reduction in the burden of malaria [25,27]. Although each of the interventions deployed during the aforementioned period might be attributed to the reduction in malaria burden observed by 2004, the contribution of each of the interventions implemented could not be fully quantified by this study.

In the CSS conducted in 2008 – 2012, the prevalence of *P. falciparum* infections dropped to <10% in Magoda and <5% in Mpapayu from 2011. Of the recent interventions, AL was introduced as first-line drug for treatment of uncomplicated malaria in January 2007and free ITNs were distributed in Magoda in 2009 through the US President Malaria Initiative (US PMI), followed by long-lasting nets in 2012 (Zuberi Mohamedi, personal communication). However, the coverage and usage of bed nets remained below 74%, i.e. far below the levels attained by 2004. It is thought-provoking that coverage and use of ITNs were much higher before 2004, when ITNs were distributed by the project compared to the most recent coverage under the national universal campaigns. Factors other than ITNs and deployment of AL could possibly be driving the most recent changes as also reported by Okiro *et al.*[34]. These include changes in climatic variables, such as rainfall and temperature, improvements of socio-economic status, health care and human land use activities, which could possibly be associated with declining malaria transmission. However, such factors were not assessed in this study and are worth taking into account in future studies.

The decline in other indices of malaria transmission intensity such as the prevalence of splenomegaly and gametocytes indicate that these areas formerly known as holo/hyper-endemic have now become low malaria transmission settings within very few years as also recently shown in the neighbouring district of Korogwe [25,27]. The shift of parasite prevalence to older children and adolescents in 2008–2012 might be attributed to delayed development of immunity among study participants due to reduced exposure to infections [57,58].

The significant decline in the prevalence of *P. falciparum* gametocytes observed in the CSS done between 2008 and 2012 could partly be attributed to the effects of ACT together with ITNs which reduce malaria transmission. Previous studies have shown that artemisinins reduce the parasite biomass drastically and thus reducing the rate of development and gametocyte carriage [59]. Such effects could be responsible for the low prevalence of gametocytes in the study areas observed in the recent CSS. Despite an increase in mean Hb levels and a decrease in the prevalence of anaemia before 2000, there were no significant changes over the study period. However, a slight increase in mean Hb and a decline in the prevalence of anaemia were observed in 2012 which could possibly indicate that there was a slight lag between the decline of malaria prevalence and downward trends of anaemia in these communities. Further monitoring will be crucial to document these important changes in malaria prevalence and their influence on other malariometric indices.

Although vector population studies have not been done in the study villages in recent years, studies conducted in the neighbouring villages in Muheza and Tanga districts showed that the density of malaria vectors has progressively declined leading to low transmission in this part of the country [60]. Furthermore, the composition of malaria vector populations in Muheza has significantly changed whereby the anthropophilic *Anopheles gambiae s.s.* have been replaced with the zoophilic *Anopheles arabiensis*[61]. These studies suggest that the changes in vector density and composition could be responsible for the low malaria burden in the area and other parts of Tanzania as recently revealed by the nation – wide malaria indicator surveys of 2011(MoH, Unpublished data). Thus, the observed trends of declining malaria burden could be linked to the interventions deployed in the study communities including ITNs, drug trials, better health care through mobile clinic, changing national malaria control policies and other unknown factors which could have indirectly caused the dramatic reduction in malaria transmission. However, the low burden of malaria observed in these villages cannot be generalised to reflect the situation in other parts of Tanzania due to high level of interventions which were deployed in the area over the study period.

## Conclusion

This study showed a marked decline of malaria burden in the study area between 1992 and 2012, initially between 1992 and 1996, and a further decline from 2008 to 2012. The decline in the initial period (1992 – 2004) was associated with the deployment of different interventions such as a weekly mobile clinic, bed nets and other research activities. However, the dramatic and steady decline observed from 2008 occurred when bed net coverage was much lower, suggesting that other factors such as introduction of ACT could also have contributed to the most recent changes. A shift of parasite prevalence from younger to older children/adolescents and increasing parasite density until 2011 imply that the transmission intensity has significantly declined leading to delayed development and low immunity among residents of these villages, making these areas which were holo/hyperendemic to become epidemics-prone or unstable malaria transmission. Further monitoring of malaria burden in these and other communities will potentially reveal future trends of malaria transmission and identify other factors which could be responsible for these significant changes of malaria epidemiology in order to appropriately devise new control strategies. The significant reduction in transmission implies the need for strengthening of the routine surveillance system and introduction of stratification to ensure that the fewer cases are captured and treated and apply focused intervention. These results also provide evidence that could be used in monitoring the progress of health related millennium development goals (MDGs).

## Competing interests

The authors declare that they have no competing interests.

## Authors’ contributions

DSI, MML, MA and ICB conceived of and designed the study; DSI, MDS, BPM and MML conducted the field work and supervised the laboratory analyses. BPM and DSI participated in data management and analysis. DSI, MA, MML and ICB wrote the manuscript. All authors read and approved the manuscript.
